# Supplementary material for: Pre-hypertrophic chondrogenic enhancer landscape of limb and axial skeleton development
Source: Nat Commun. 2024 Jun 6;15:4820. doi: 10.1038/s41467-024-49203-2 (PMC11156918; doi:10.1038/s41467-024-49203-2)
Supplement: Supplementary file 1 — Supplementary Information [file 41467_2024_49203_MOESM1_ESM.pdf]

Supplementary Info To

Pre-Hypertrophic Chondrogenic Enhancer Landscape of Limb and Axial Skeleton Development

Fabrice Darbellay[1,2,3], Anna Ramisch[4], Lucille Lopez-Delisle[5], Michael Kosicki[3], Antonella Rauseo[1,2], Zahra Jouini[1,2], Axel Visel[3,6,7] and Guillaume Andrey[1,2]

Affiliations

[1]Department of Genetic Medicine and Development, Faculty of Medicine, University of Geneva, 1211 Geneva, Switzerland

[2]Institute of Genetics and Genomics in Geneva (iGE3), University of Geneva, 1211 Geneva, Switzerland

[3]Environmental Genomics and Systems Biology Division, Lawrence Berkeley Laboratory, CA 94720, USA

[4]Department of Basic Neurosciences, Faculty of Medicine, University of Geneva, 1211 Geneva, Switzerland

[5]School of Life Sciences, Ecole Polytechnique Fédérale de Lausanne (EPFL), 1015 Lausanne, Switzerland

[6]U.S. Department of Energy Joint Genome Institute, Lawrence Berkeley Laboratory, CA 94720, USA

[7]School of Natural Sciences, University of California, Merced, CA 95343, USA

Correspondence should be addressed to G.A. (email: guillaume.andrey@unige.ch).

Supplementary Figure 1

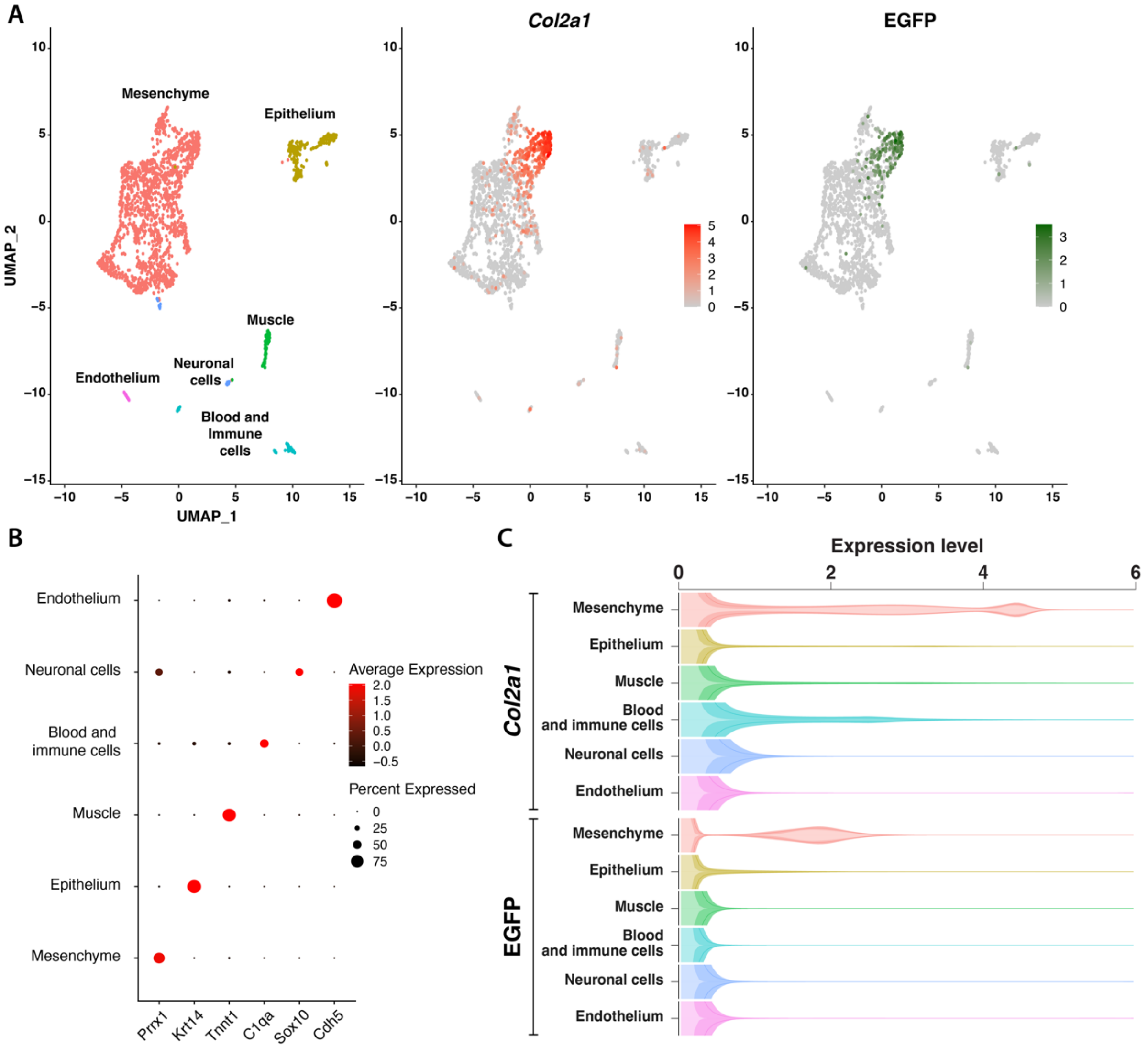

Supplementary Figure 1: Single-cell analysis of E14.5 *Col2a1*$^{EGFP/EGFP}$ limbs. **A.** Left panel: UMAP visualization of clustering of E14.5 limb cells. Middle: expression of *Col2a1* across the UMAP. Right: Expression of EGFP across the UMAP. **B.** Selected marker gene expression per cluster. **C.** Expression distribution of *Col2a1* and EGFP per limb cluster estimated by baredSC (*1*). Line shows mean and shaded area around the line indicates the 68% confidence interval. Density values, displayed on the y axis, were truncated at 0.3 and mirrored. Note the strong expression of both genes in the mesenchyme cluster.

Supplementary Figure 2

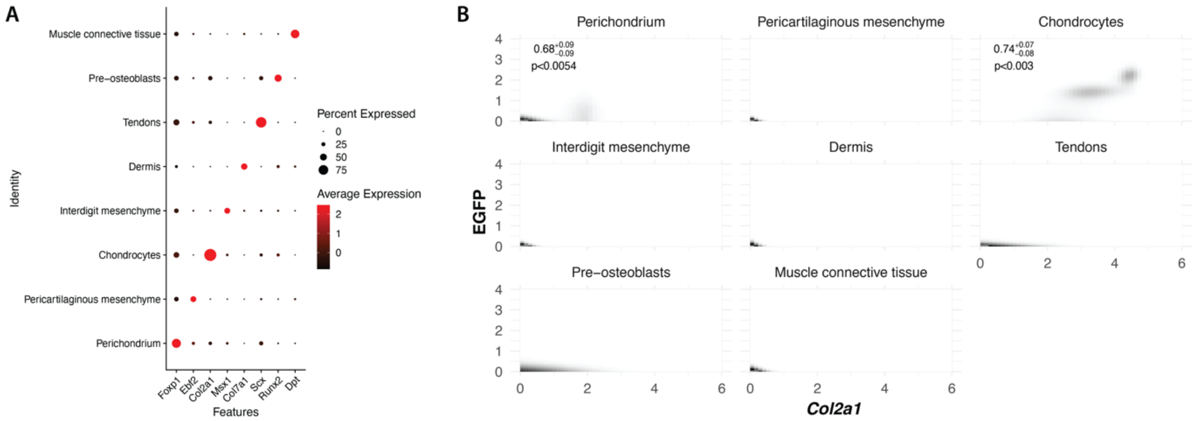

Supplementary Figure 2: A. Selected marker gene expression per mesenchymal cluster. **B.** Co-expression of *Col2a1* (x-axis) and EGFP (y-axis) in all limb mesenchymal clusters as determined by baredSC (*1*). For clusters with fraction of cells expressing *Col2a1* above 2, correlation coefficient is displayed with confidence interval and estimated p-value.

Supplementary Figure 3

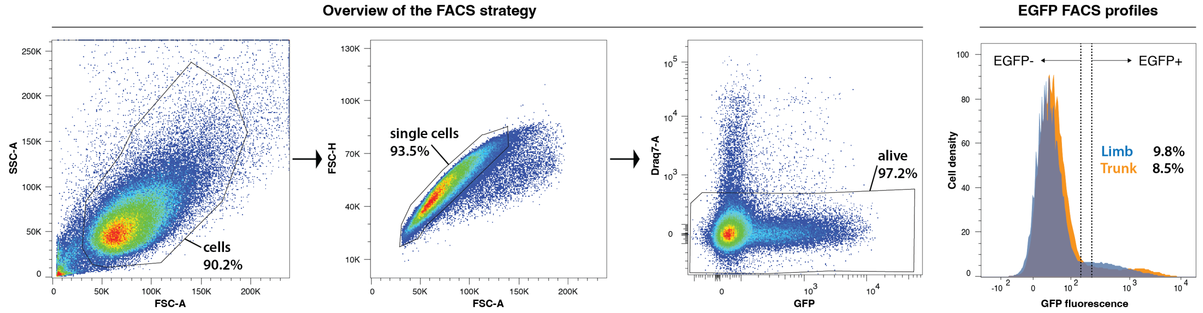

Supplementary Figure 3: Graphical illustration of the FACS strategy followed to isolate EGFP+ and EGFP- cell population (E14.5 *Col2a1*^{EGFP} limbs) (three left panels) and GFP fluorescence profiles of E14.5 *Col2a1*^{EGFP} limbs and trunks (most right panel).

Supplementary Figure 4

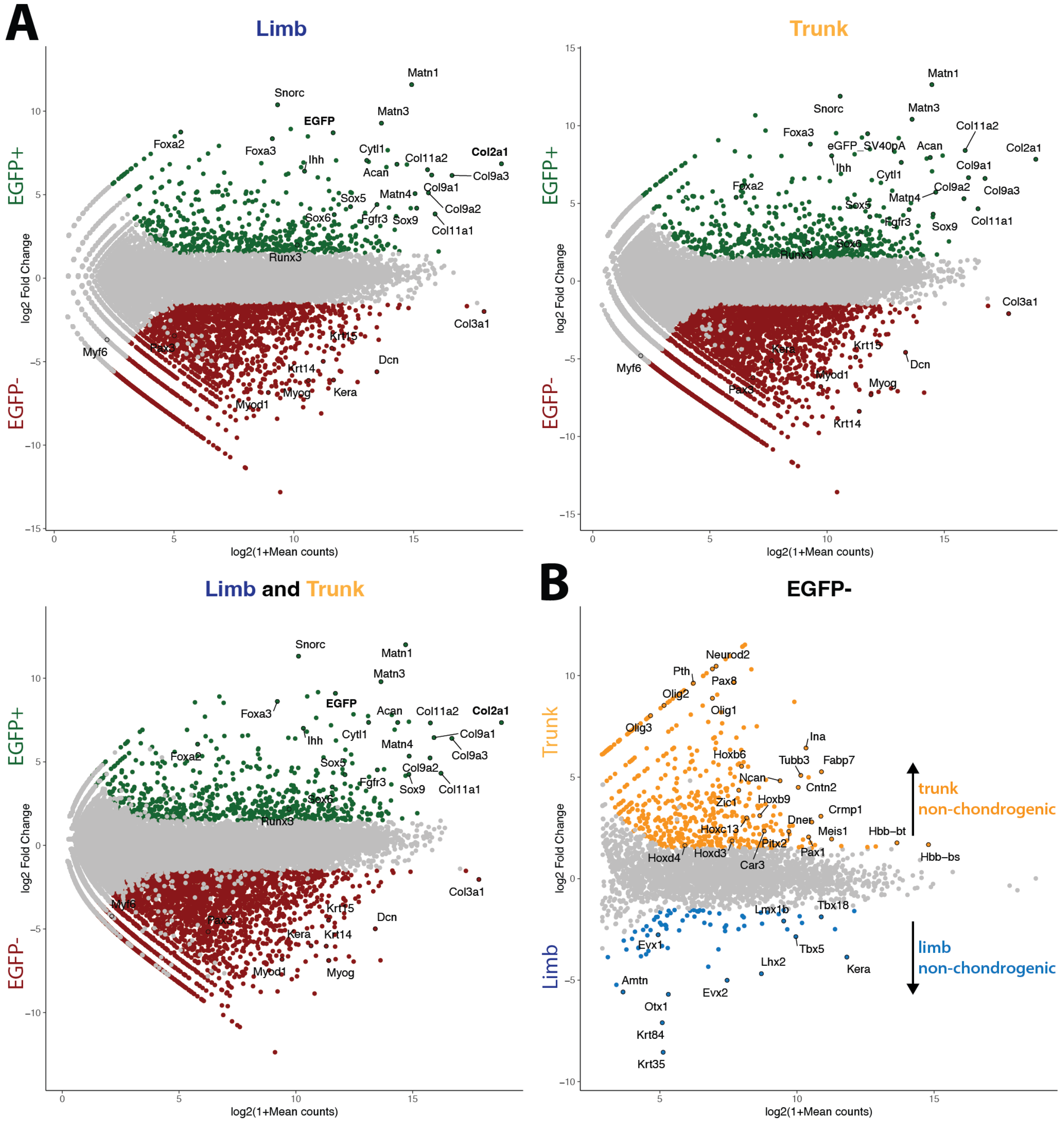

Supplementary Figure 4: A. Enriched genes in EGFP+ (green) and EGFP- (red) cell populations in limbs (top left), trunk (top right) and combined datasets (bottom left). **B.** Non-chondrogenic (EGFP-) marker genes with an expression preference in limb (blue) or trunk (orange). Statistical test used: DESeq2 Wald test, differential expression was scored when abs(log_2FC)>1.5 and FDR-corrected p-value<0.05.

Supplementary Figure 5

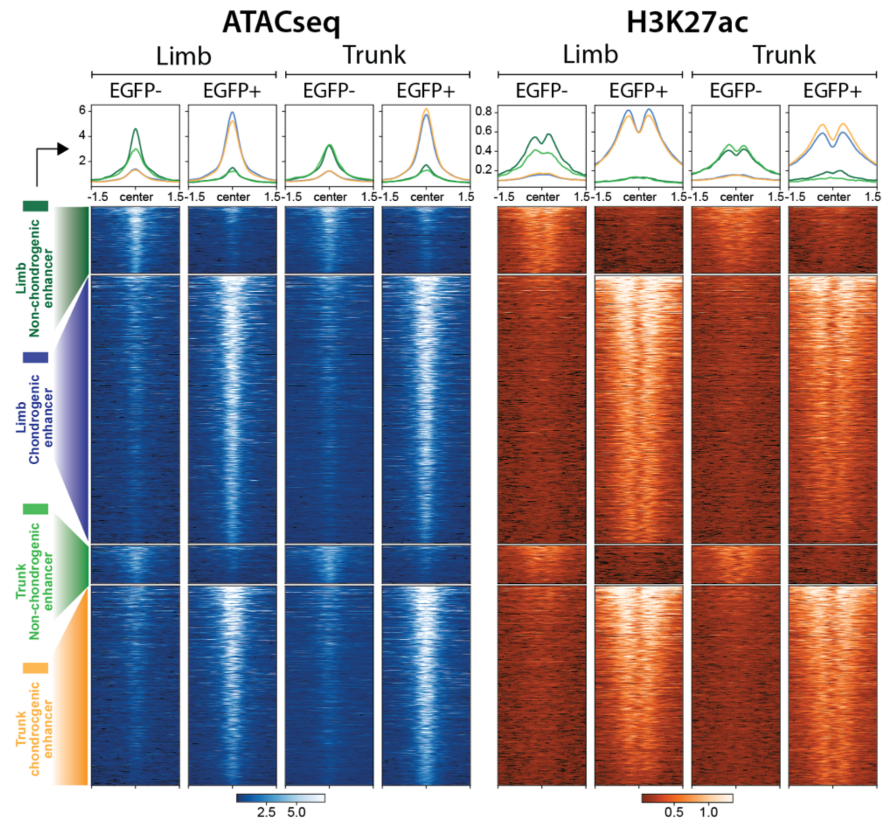

Supplementary Figure 5: Analyses of chondrogenic chromatin landscapes and enhancers. ATAC-seq and H3K27ac coverages over 3kb for limb and trunk chondrogenic and non-chondrogenic enhancers are centered at the corresponding merged ATAC-seq peaks located within a 75bp window. Enhancers might be present in multiple categories.

Supplementary Figure 6

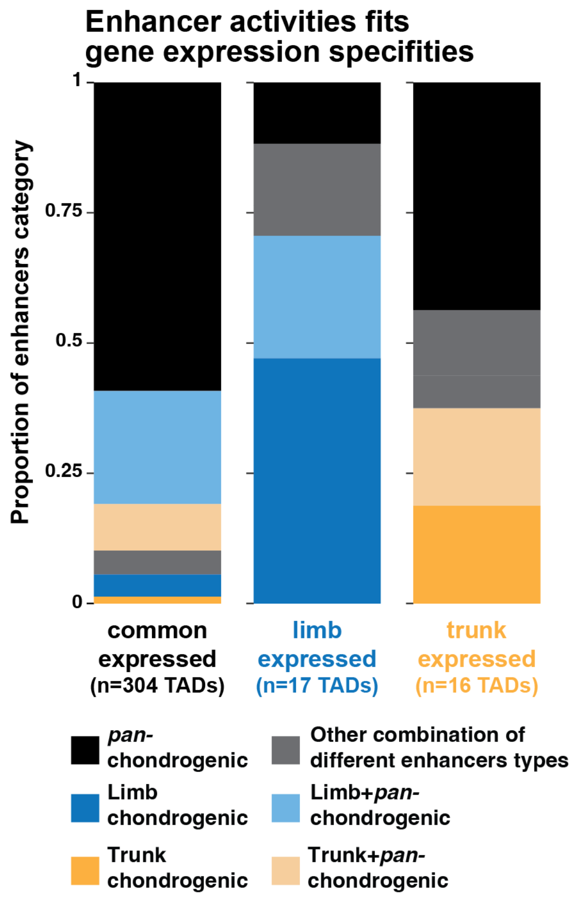

Supplementary Figure 6: Association between trunk- and limb- preference of chondrogenic protein coding genes and chondrogenic enhancers. Proportion of TADs containing the different enhancer types: only *pan*-chondrogenic enhancers (active in both limb and trunk chondrocytes), only limb-enriched enhancers (active preferentially in limb chondrocytes), only trunk-enriched enhancers (active preferentially in trunk chondrocytes) and combinations thereof split by TADs containing common-, limb-, or trunk-expressed genes. Note that the proportion of limb and trunk enhancers is increased in limb or trunk-associated TADs.

Supplementary Figure 7

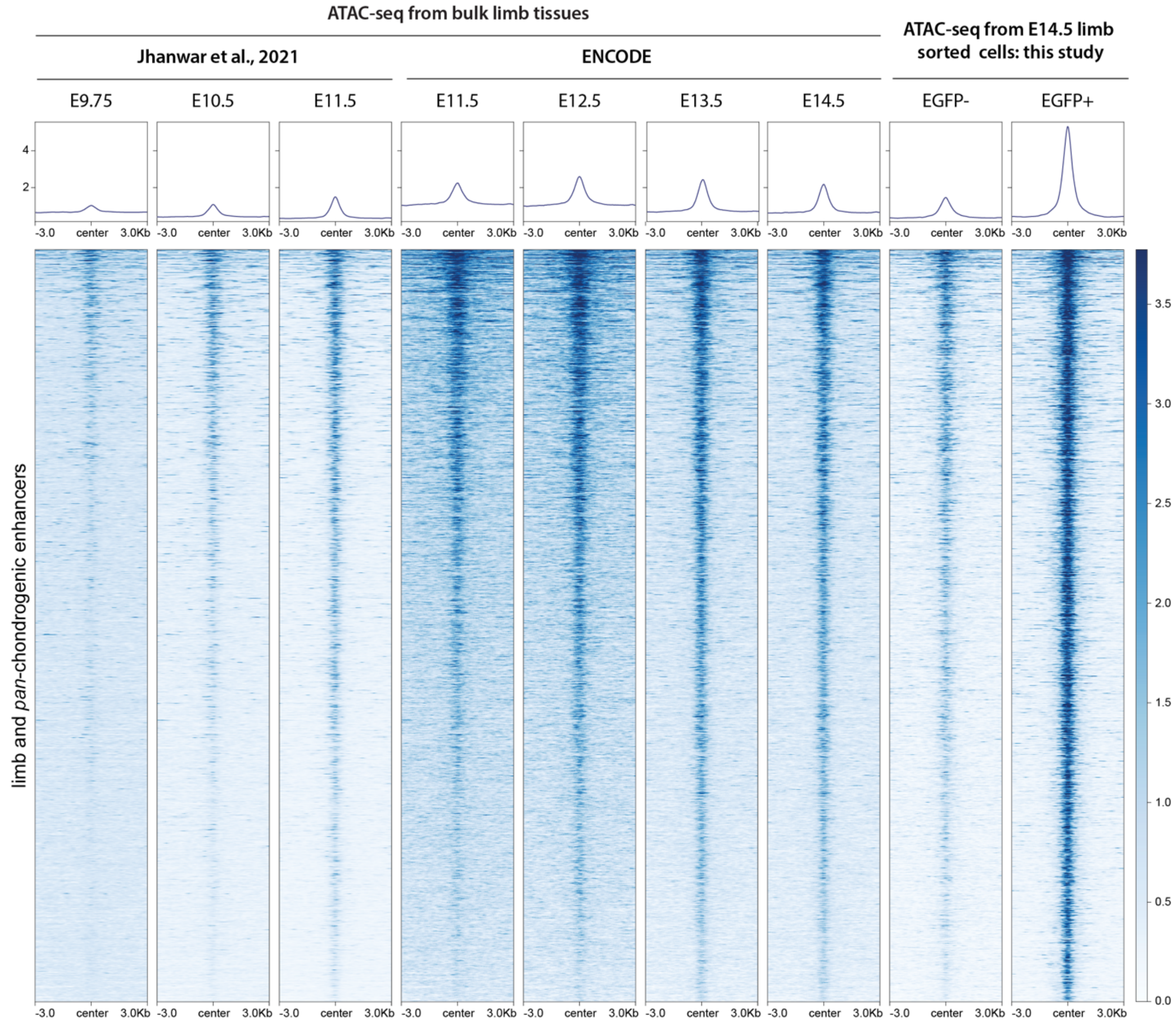

Supplementary Figure 7: ATAC-seq signal at chondrogenic enhancers over developmental time. At E9.75 only a few regions display signal while at E11.5 the maximum signal is obtained (to compare with E14.5 bulk). ATAC-seq coverages over 6kb are centered at the corresponding merged ATAC-seq peaks located within a 75bp window.

Supplementary Figure 8

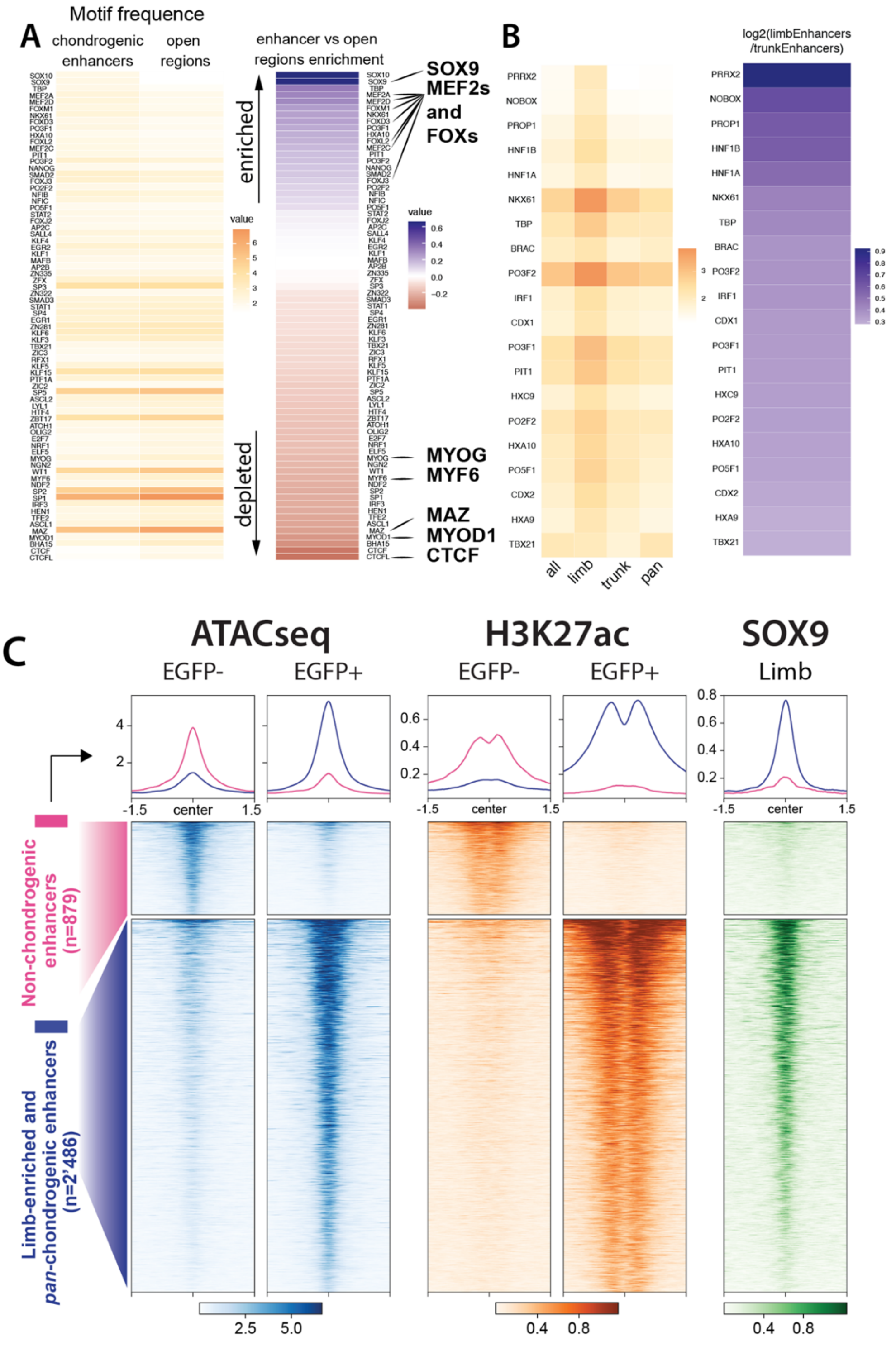

Supplementary Figure 8: Transcription factor binding at chondrogenic enhancers. **A.** Left: All enriched motifs between chondrogenic enhancers and "*inactive*" accessible regions. Right: ratio of motifs enrichment between chondrogenic enhancers and "*inactive*" accessible regions. **B.** PRXX2 motif enriched in limb-enriched chondrogenic enhancers. **C.** Binding of *SOX9* at limb-enriched and *pan*-chondrogenic enhancers (n=2'486) compared to non-chondrogenic enhancers enrichment (n=879). Heatmaps are generated from the limb EGFP+ and EGFP- datasets. ATAC-seq, H3K27ac and SOX9 coverages over 3kb centered at the corresponding merged ATAC-seq peaks located within a 75bp window.

Supplementary Figure 9

A

Span_TAD_Castro_mm39	size (bp)	chondrogenic genes	chondrogenic genes [n]	chondrogenic enhancers [n]
chr5:41207343-41967343	760000	Nkx3-2	1	3
chr10:77815834-86415864	8600030	Aire, Chst11, Fstl3, Odf3l2, Slc41a2	5	18
chr18:58093072-59213072	1120000			3
chr13:47213476-49893476	2680000	Barx1, Ecm2, Id4, Ninj1, Omd, Rnf144b	6	13
chr1:57999159-59119159	1120000			
chr15:40993396-42793396	1800000			
chr6:119426961-120466961	1040000	B4galnt3, Ninj2	2	3
chr11:111430826-113510826	2080000	Sox9	1	33
-	**19200030**		**15**	**73**

B

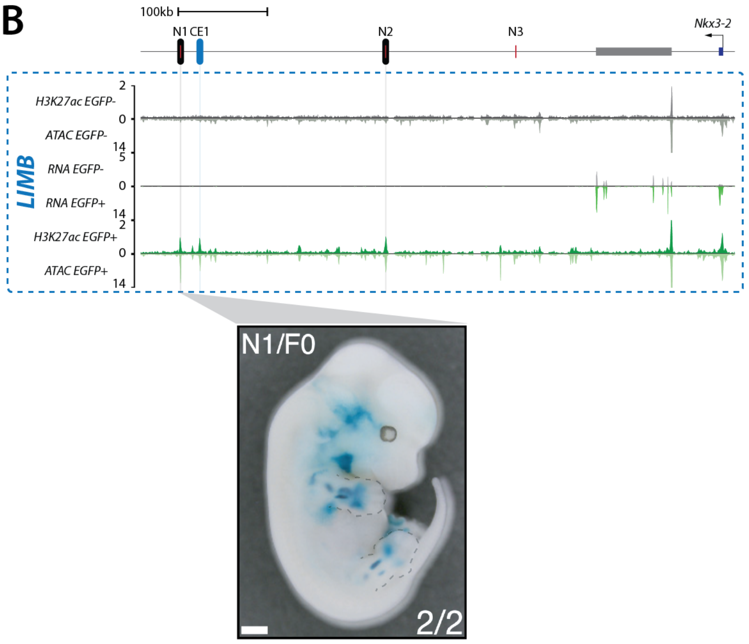

Supplementary Figure 9: A. Eight loci under selection were identified in Longshanks mice, six of which contain numerous chondrogenic genes and enhancers (*2*). **B.** Distribution of RNA-seq, ATAC-seq and H3K27ac ChIP-seq normalized coverages in limb (blue box) EGFP+ and EGFP- cells at the *Nkx3-2* loci. EGFP+ datasets are colored in green, EGFP- datasets in grey. RNA-seq and ATAC-seq coverages are an average of two replicates. Vertical bars highlight the genomic position of chondrogenic enhancers. Protein-coding genes are represented as grey or blue boxes. At the *Nkx3-2* loci, we predicted 3 chondrogenic enhancers: 2 are categorized as *pan*-chondrogenic (black ovals) and 1 is limb-enriched (CE1, blue oval). 2 of the 3 chondrogenic enhancers have been identified as potentially affecting tibial length in Longshanks mice (N1 and N2 from (*2*)). The N3 element is also highlighted but was not predicted to be chondrogenic-specific. Photo was obtained from (*2*).

Supplementary Figure 10

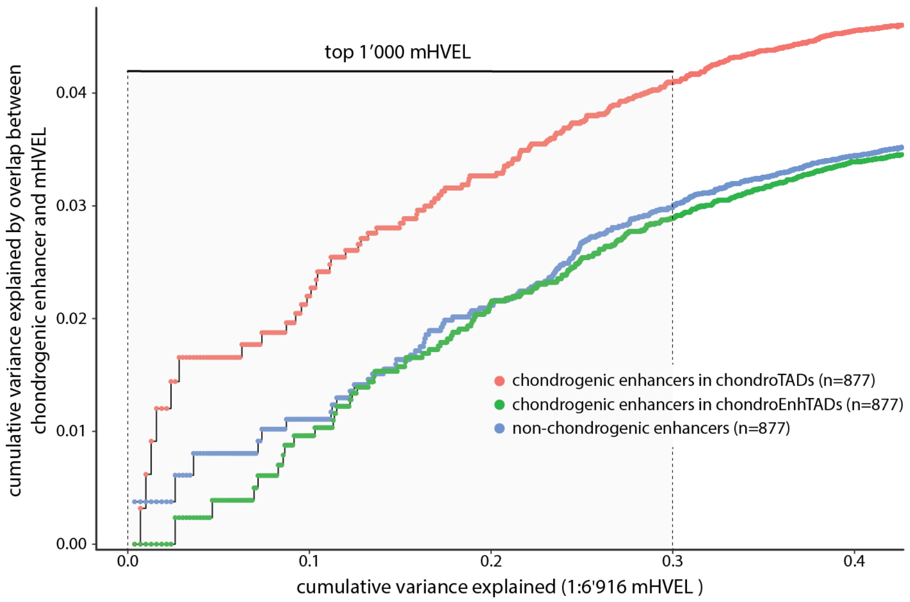

Supplementary Figure 10: Cumulative height variance explained by the 6'916 mHVEL (x-axis, regions sorted from highest to lowest variance) and the same variance explained by the enhancers overlapping the SNP-loci (y-axis). Enhancers on the X and Y chromosome were excluded from the analysis.

Supplementary Figure 11

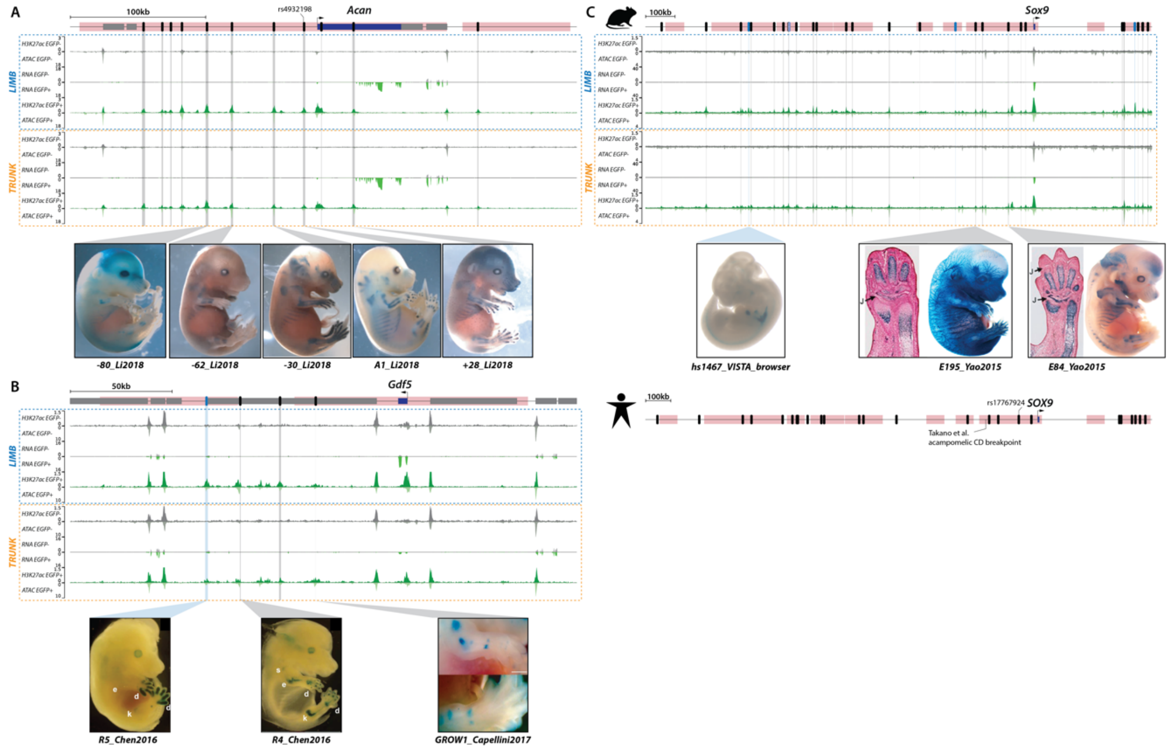

Supplementary Figure 11: A-B-C Distribution of RNA-seq, ATAC-seq and H3K27ac ChIP-seq normalized coverages in limb (blue box) and trunk (orange box) EGFP+ and EGFP- cells at the *Acan*, *Sox9* and *Gdf5* loci**.** mHVEL are highlighted in pink (*3*). EGFP+ datasets are colored in green, EGFP- datasets in grey. RNA-seq and ATAC-seq coverages are an average of two replicates. Vertical bars highlight the genomic position of chondrogenic enhancers. Protein-coding genes are represented as grey or blue boxes. **A.** 12 chondrogenic enhancers are predicted at the *Acan* locus and categorized as *pan*-chondrogenic (black ovals). 6 out of the 12 enhancers have already been shown to drive chondrogenic expression of marker genes. Photos were obtained from (*4*). 1 human SNP associated by Yengo et al. with height was identified in the enhancer A1 (*3*). **B.** 4 chondrogenic enhancers are predicted at the *Gdf5* locus: 3 are categorized as *pan*-chondrogenic (black ovals) and 1 is limb-enriched (blue oval). 3 out of the 4 enhancers have already been shown to drive chondrogenic expression of marker genes. Photos were obtained from (*5, 6*). Shoulder (s), elbow (e), knee (k) and digits (d) are identified on the R4 and R5 photos. **C.** 29 chondrogenic enhancers are predicted at the mouse *Sox9* locus, 25 are categorized as *pan*-chondrogenic (black ovals) and 4 are limb-enriched (blue ovals). 3 out of the 29 enhancers have already been shown to drive chondrogenic expression of marker genes. Photos of E195 and E84 were obtained from (*7*) and from the VISTA Enhancer browser for hs1467 (*8*). Hs1467 is published by Osterwalder et al. (*9*). 1 human SNP associated by Yengo et al. with height was identified in the enhancer E84 (*3*). Carpal and phalangeal joints (J) are identified in the E84 and E195 sections. Below the human *SOX9* locus with lift over chondrogenic enhancers and the breakpoint involved in acampomelic CD (*10*).

Supplementary Figure 12

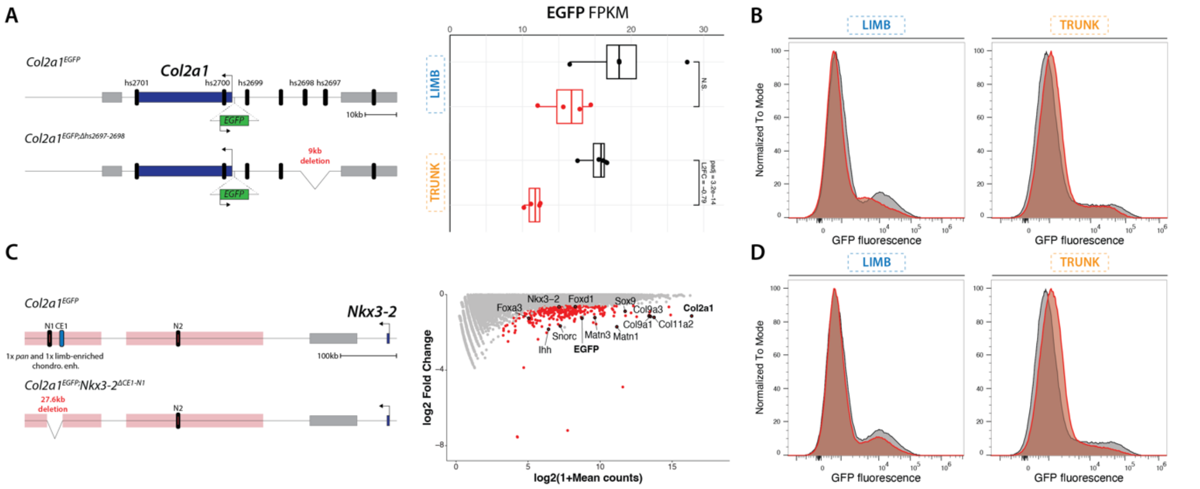

Supplementary Figure 12: A. Schematic representation of *Col2a1*$^{EGFP;\Delta hs2697\text{-}2698}$ and boxplots showing the distribution of EGFP FPKMs obtained from *Col2a1*^{EGFP} (black) or *Col2a1*$^{EGFP;\Delta hs2697\text{-}2698}$ (red) E14.5 limbs and trunk (blue or orange). In boxplot, boxes indicate the first and third quartiles, the whiskers indicate ±1.5 × interquartile range and the horizontal line within the boxes indicates the median. Statistical test used: DESeq2 Wald test, padj is the FDR-corrected by Benjamini-Hochberg method two-tailed p-value, and L2FC is the $\log_2$ fold change estimated by DESeq2 on the gene raw counts. N=4 biologically independent samples per condition. Source data are provided as a Source Data file. **B.** Profiling of GFP signal by flow cytometry in *Col2a1*^{EGFP} (black) or *Col2a1*$^{EGFP;\Delta hs2697\text{-}2698}$ (red) bulk E14.5 trunk or limb tissues. **C.** Schematic representation *Col2a1*^{EGFP}*;Nkx3-2*$^{\Delta CE3\text{-}N1}$ and relevant chondrogenic genes significantly reduced in *Col2a1*^{EGFP}*;Nkx3-2*$^{\Delta CE1\text{-}N1}$ E14.5 trunk. Non-coding mHVEL are highlighted in pink. Statistical test used: DESeq2 Wald test, significant differential expression (reduction) was highlighted in red when ($\log_2$FC)<-0.6, a value similar to the $\log_2$FC observed for *Nkx3-2*, and FDR-corrected by Benjamini-Hochberg method two-tailed p-value<0.05. Protein-coding genes are represented as grey or blue boxes. **D.** Profiling of GFP signal by flow cytometry in *Col2a1*^{EGFP} (black) or *Col2a1*^{EGFP}*;Nkx3-2*$^{\Delta CE1\text{-}N1}$ bulk E14.5 trunk or limb tissues.

References

1. L. Lopez-Delisle, J.-B. Delisle, baredSC: Bayesian approach to retrieve expression distribution of single-cell data. *BMC Bioinformatics* **23**, (2022).
2. J. P. Castro *et al.*, An integrative genomic analysis of the Longshanks selection experiment for longer limbs in mice. *eLife* **8**, (2019).
3. L. Yengo *et al.*, A saturated map of common genetic variants associated with human height. *Nature* **610**, 704-712 (2022).
4. I. M. H. Li *et al.*, Differential tissue specific, temporal and spatial expression patterns of the Aggrecan gene is modulated by independent enhancer elements. *Scientific reports* **8**, 950 (2018).
5. T. D. Capellini *et al.*, Ancient selection for derived alleles at a GDF5 enhancer influencing human growth and osteoarthritis risk. *Nat Genet* **49**, 1202-1210 (2017).
6. H. Chen *et al.*, Heads, Shoulders, Elbows, Knees, and Toes: Modular Gdf5 Enhancers Control Different Joints in the Vertebrate Skeleton. *PLoS Genet* **12**, e1006454 (2016).
7. B. Yao *et al.*, The SOX9 upstream region prone to chromosomal aberrations causing campomelic dysplasia contains multiple cartilage enhancers. *Nucleic acids research* **43**, 5394-5408 (2015).
8. A. Visel, S. Minovitsky, I. Dubchak, L. A. Pennacchio, VISTA Enhancer Browser--a database of tissue-specific human enhancers. *Nucleic acids research* **35**, D88-92 (2007).
9. M. Osterwalder *et al.*, Enhancer redundancy provides phenotypic robustness in mammalian development. *Nature* **554**, 239-243 (2018).
10. T. Takano *et al.*, Adult acampomelic campomelic dysplasia and disorders of sex development due to a reciprocal translocation involving chromosome 17q24.3 upstream of the SOX9 gene. *Eur J Med Genet* **64**, 104332 (2021).
