# Supplementary material for: Pre-hypertrophic chondrogenic enhancer landscape of limb and axial skeleton development
Source: Nat Commun. 2024 Jun 6;15:4820. doi: 10.1038/s41467-024-49203-2 (PMC11156918; doi:10.1038/s41467-024-49203-2)
Supplement: Supplementary file 3 — Description of Additional Supplementary Files [file 41467_2024_49203_MOESM3_ESM.pdf]

Description of Additional Supplementary Files

File Name: Supplementary Data 1
Description: scRNA-seq marker genes for all clusters. Two-sided Wilcoxon Rank Sum test, p-value adjustment is performed using Bonferroni correction based on the total number of genes in the dataset.

File Name: Supplementary Data 2
Description: scRNA-seq marker genes in reclustered mesenchymal cells. Two-sided Wilcoxon Rank Sum test, p-value adjustment is performed using Bonferroni correction based on the total number of genes in the dataset.

File Name: Supplementary Data 3
Description: List of chondrogenic genes and their assigned tissue specificity (mm39). Chondrogenic marker genes displaying a significant expression preference in limb or trunk EGFP+ cells. Statistical test used: DESeq2 Wald test, limb or trunk-specific expression was scored when abs(log2FC)>1.5 and FDR-corrected by Benjamini-Hochberg method two-tailed p-adj<0.05.

File Name: Supplementary Data 4
Description: List of non-chondrogenic genes and their assigned tissue specificity (mm39). Non-chondrogenic marker genes displaying a significant expression preference in limb or trunk EGFP- cells. Statistical test used: DESeq2 Wald test, limb or trunk-specific expression was scored when abs(log2FC)>1.5 and FDR-corrected by Benjamini-Hochberg method two-tailed p-adj<0.05.

File Name: Supplementary Data 5
Description: Coordinates of chondrogenic enhancers and their assigned tissue specificity (mm39).

File Name: Supplementary Data 6
Description: Coordinates of non-chondrogenic enhancers (mm39).

File Name: Supplementary Data 7
Description: Coordinates of ChondroTAD (mm39).

File Name: Supplementary Data 8
Description: List of protein-coding chondrogenic genes located in chondroTAD.

File Name: Supplementary Data 9
Description: Coordinates of chondrogenic enhancers located in chondroTAD (mm39).

File Name: Supplementary Data 10
Description: Coordinates of ChondroEnhTAD (mm39).

File Name: Supplementary Data 11
Description: Coordinates of chondrogenic enhancers located in chondroEnhTAD (mm39).

File Name: Supplementary Data 12
Description: List and coordinates of protein-coding genes located in chondroTAD (mm39).

File Name: Supplementary Data 13
Description: Gene Ontology results for protein-coding genes located in chondroTADs.

File Name: Supplementary Data 14
Description: List and coordinates of protein-coding genes located in chondroEnhTAD (mm39).

File Name: Supplementary Data 15
Description: Gene Ontology results for protein-coding genes located in chondroEnhTADs.

File Name: Supplementary Data 16
Description: Coordinates of SOX9 ChIP-seq peaks remapped from Yamashita et al. (mm39).

File Name: Supplementary Data 17
Description: Overlap between human height cumulative variance, mHVEL and mouse chondrogenic enhancers.

File Name: Supplementary Data 18
Description: Combined results of the differential expression analyses presented in Fig. 5. DESeq2 results (l2fc is log2 fold-change and padj is the FDR-corrected by Benjamini-Hochberg method two-tailed p-value) are reported for each DESeq2 comparison performed between E14.5 limb or trunk samples. Del(Col2a1_CE), del(Fgfr3_CE), del(Hhip_CE) and del(Nkx3-2_CE) corresponds respectively to $Col2a1^{EGFP;\Delta hs2697\text{-}2698}$, $Col2a1^{EGFP};Fgfr3^{\Delta hs2696}$, $Col2a1^{EGFP};Hhip^{\Delta CE2\text{-}3}$ and $Col2a1^{EGFP};Nkx3\text{-}2^{\Delta CE1\text{-}N1}$. Control is $Col2a1^{EGFP}$. Individual FPKM values are also reported for each sample.

File Name: Supplementary Data 19
Description: Coordinates of LiftOver mouse chondrogenic enhancer in human genome (hg38).

File Name: Supplementary Data 20
Description: Genotyping oligonucleotides used in this study.

File Name: Supplementary Data 21
Description: Enhancer assay additional information. VISTA name, coordinates hg(38), cloning oligonucleotides and sequences.

File Name: Supplementary Movie 1
Description: Three-dimensional rendering of the GFP signal obtained by light-sheet imaging of an E14.5 $Col2a1^{EGFP}$ fetus. Forelimb is colored in red, hindlimb in green and trunk skeletal elements in grey. Only the right-side of the fetus has been reconstructed. The video scale bar is dynamic, with size ranging from 800 to 1000μm.
