# Supplementary material for: Pre-hypertrophic chondrogenic enhancer landscape of limb and axial skeleton development
Source: Nat Commun. 2024 Jun 6;15:4820. doi: 10.1038/s41467-024-49203-2 (PMC11156918; doi:10.1038/s41467-024-49203-2)
Supplement: Supplementary file 6 — Reporting Summary [file 41467_2024_49203_MOESM6_ESM.pdf]

nature portfolio

Corresponding author(s): Guillaume Andrey

Last updated by author(s): 11.04.2024

Reporting Summary

Nature Portfolio wishes to improve the reproducibility of the work that we publish. This form provides structure for consistency and transparency in reporting. For further information on Nature Portfolio policies, see our Editorial Policies and the Editorial Policy Checklist.

Statistics

For all statistical analyses, confirm that the following items are present in the figure legend, table legend, main text, or Methods section.

n/a	Confirmed	
☐	☒	The exact sample size (*n*) for each experimental group/condition, given as a discrete number and unit of measurement
☐	☒	A statement on whether measurements were taken from distinct samples or whether the same sample was measured repeatedly
☐	☒	The statistical test(s) used AND whether they are one- or two-sided<br>*Only common tests should be described solely by name; describe more complex techniques in the Methods section.*
☒	☐	A description of all covariates tested
☐	☒	A description of any assumptions or corrections, such as tests of normality and adjustment for multiple comparisons
☐	☒	A full description of the statistical parameters including central tendency (e.g. means) or other basic estimates (e.g. regression coefficient) AND variation (e.g. standard deviation) or associated estimates of uncertainty (e.g. confidence intervals)
☐	☒	For null hypothesis testing, the test statistic (e.g. *F*, *t*, *r*) with confidence intervals, effect sizes, degrees of freedom and *P* value noted<br>*Give P values as exact values whenever suitable.*
☒	☐	For Bayesian analysis, information on the choice of priors and Markov chain Monte Carlo settings
☒	☐	For hierarchical and complex designs, identification of the appropriate level for tests and full reporting of outcomes
☒	☐	Estimates of effect sizes (e.g. Cohen's *d*, Pearson's *r*), indicating how they were calculated

Our web collection on statistics for biologists contains articles on many of the points above.

Software and code

Policy information about availability of computer code

Data collection	No commercial, open source and custom code was used to collect the data in this study.
Data analysis	The NGS datasets presented here were mapped either on GRCm39/mm39 or on a customized GRCm39/mm39_eGFP-SV40pA genome. The GTF annotations used in this work derive from ENSEMBL GRCm39 release 104 and are filtered against read-through/overlapping transcripts, keeping only transcripts annotated as 'protein-coding' for 'protein-coding' genes, thus discarding transcripts flagged as 'retained_intron', 'nonsense-mediated decay' etc., to conserve only non-ambiguous exons and avoid quantitative bias during data analysis by STAR/Cufflinks. GRCm39/mm39_eGFP-SV40pA sequence, the filtered GTF file and the scripts used to construct it are available on Zenodo (https://doi.org/10.5281/zenodo.7837435).<br><br>1- scRNA-seq.<br>Reads were mapped to a customized GRCm39/mm39_eGFP-SV40pA reference genome and corresponding gene annotation as for the bulk RNA-seq using 10X Genomics Cell Ranger v6.1.2 and data analyzed with the R package Seurat v4.3.0. Briefly, Cell Ranger filtered_feature_bc_matrix.h5 matrix was first imported into Seurat (min.cells = 3, min.features = 200), filtered (nFeature_RNA > 200 & nFeature_RNA < 5000 & percent.mt < 5 & nCount_RNA > 1000 & nCount_RNA < 26000), log-normalized and scaled. The reporter gene eGFP-2SV40pA was excluded from the list of variable gene in all subsequent normalizations. Scaled data were then used for principal component analysis (PCA) with npcs=100 and non-linear dimensional reduction by Uniform Manifold Approximation Projection (UMAP) ndims=1:100 as input. We then identified and excluded doublets using the R package DoubletFinder v2.0.3 (PCs = 1:100, pN = 0.25, pK =0.07 , nExp =55 , reuse.pANN = FALSE, sct = FALSE). Cells were then further filtered to exclude blood cell present in our dataset (percent.mt > 1 & percent.mt < 5). We then applied a first SCTransform normalization on our dataset, scored the cell-cycle and performed a second SCTransform normalization to regress it out. Following this regression, cells were then clustered using PCA (npcs=100), UMAP (dims=1:100) and nearest neighbors of each cell were calculated (dims=1:100). Clusters were determined using Seurat FindClusters function with default

parameters and a resolution of 1.1. In that way 16 clusters were defined (n=2041 cells). Identification of clusters identity was done by using Seurat FindAllMarkers on the RNA assay and mesenchyme clusters were then merged. The list of marker genes is provided in Supplementary Table 1. Since the interest of this work was focus on the mesenchymal populations of cells that express Col2a1, we then subsetted and re-clustered the mesenchyme cluster alone (n=1617 cells). To do so we repeated for the subsetted cells a SCTransform normalization regressing out the cell-cycle and perfomed PCA with npcs=50. UMAP embedding was calculated with ndims=1:50 and cluster resolution was set at 0.8 after finding neighbors with dims=1:50 to reveal subpopulations. We observed 8 mesenchyme subpopulations that we named according to their marker genes. Identity markers were found using FindAllMarkers on the RNA assay and are provided in Supplementary Table 2. DotPlots and FeaturesPlots were generated from the RNA assay of the Seurat objects. To correct the distribution of expression from sampling noise, we used baredSC v1.0.0 (--minNeff 200 --xmax 6). This allows us to evaluate a 68% confidence interval on the expression distribution per cluster. BaredSC was also used to compute the co-expression distribution per cluster in Supplementary Figure S2B where correlation was given with 68% confidence interval and p-value indicated is the mean probability + estimated standard deviation on the mean probability. The joint density UMAP was produced using the R packages Nebulosa v1.6.0 and scCustomize v1.1.0 .

2- ATAC-seq.
ATAC-seq datasets were analyzed following . NextSeq adapter sequences and bad quality bases were removed using CutAdapt v1.18 (-a CTGTCTCTTATACACATCTCCGAGCCCACGAGAC -A CTGTCTCTTATACACATCTGACGCTGCCGACGA -q30 -m15). Reads were then mapped to GRCm39/mm39 using Bowtie2 v2.3.5.1 (--very-sensitive --no-unal --no-mixed --no-discordant --dovetail -X 1000). Reads with mapping quality below 30, mapping to mitochondria, or not properly paired were removed from the analysis with Samtools view v1.10 . PCR duplicates were filtered using Picard v2.21.1 (https://github.com/broadinstitute/picard). BAM file was converted to BED with Bedtools v2.28.0 . Peak calling and coverage was done using MACS2 v2.2.7.1 (callpeak --nomodel --call-summits --extsize 200 --shift -100 --keep-dup all). Coverage was normalized by the number of millions of reads falling into MACS2 summits +/- 500bp using Bedtools v2.28.0 . When indicated, coverage profiles represent an average of the normalized coverage of all replicates.

3- ChIP-seq.
A similar number of reads (39x10e6) were randomly sampled from each ChIP-seq dataset to correct for sequencing depth variation using Seqtk v1.3 (-s 100) (https://github.com/lh3/seqtk). TruSeq adapter sequences and bad quality bases were removed using CutAdapt v1.18 (-a AGATCGGAAGAGCACACGTCTGAACTCCAGTCAC -q30 -m15). Reads were then mapped to GRCm39/mm39 using Bowtie2 v2.3.5.1 with default parameters. Reads were then filtered for a MAPQ≥30 using Samtools view v1.10 and the coverage and peak calling was obtained after extension of the reads by 200 bp using MACS2 v2.2.7.1 (callpeak --nomodel --call-summits --extsize 200). Coverage was normalized by the number of million tags used by MACS2.

4- RNA-seq.
For cDNA libraries generated from FACS sorted cells, NextSeq adapter sequences and bad quality bases were removed using CutAdapt v1.18 (90) (-a CTGTCTCTTATACACATCTCCGAGCCCACGAGAC -q30 -m15). Unstranded reads were then mapped to both GRCm39/mm39 (for coverage visualization presented in Figure 4, Supplementary Fig. S8 and S9) and to a customized GRCm39/mm39_eGFP-SV40pA for differential gene expression analysis. We used STAR v2.7.2b with the filtered GTFs (see Genomic data section) allowing to get a gene quantification simultaneously (--outSAMstrandField intronMotif --sjdbOverhang '99' --sjdbGTFfile $gtfFile --quantMode GeneCounts --outFilterType BySJout --outFilterMultimapNmax 20 --outFilterMismatchNmax 999 --outFilterMismatchNoverReadLmax 0.04 --alignIntronMin 20 --alignIntronMax 1000000 --alignMatesGapMax 1000000 --alignSJoverhangMin 8 --alignSJDBoverhangMin 1). Gene expression computations were performed using uniquely mapping reads extracted from STAR alignments and genomic annotations from filtered GTF (see Genomic data section). FPKM values were determined by Cufflinks v2.2.1 (--max-bundle-length 10000000 --multi-read-correct --library-type "fr-unstranded" --no-effective-length-correction -M MTmouse.gtf). Coverage was computed with Bedtools v2.28.0 using uniquely mapped reads (NH:i:1 tag). When indicated, coverage profiles represent an average of the replicates. This was done by dividing each replicate by the number of millions of uniquely mapped reads (for normalization) and calculating the average coverage. Differentially expressed genes were tested using the R package DESeq2 v1.34.0 . Chondrogenic (EGFP+) and non-chondrogenic (EGFP-) marker genes were defined from the DESeq2 Wald test results with thresholds being respectively set at (log2FC)>1.5 and (log2FC)<1.5 with FDR-corrected p-value<0.05. Bulk stranded cDNA libraries were processed with the following differences. TruSeq adapter sequences instead of Nextera sequences were removed using CutAdapt (-a AGATCGGAAGAGCACACGTCTGAACTCCAGTCAC -q30 -m15). At the mapping step, the option --outSAMstrandField intronMotif was removed and the library type was set to "fr-firststrand" instead of "fr-unstrand" in Cufflinks. For differentially expressed genes no threshold was applied on the log2FC.

5- Genomic tracks visualizations.
ATAC-seq, ChIP-seq and RNA-seq tracks presented in the figures were generate using pyGenomeTracks v3.8.

6- Identification of enhancers.
Bedtools merge and intersect v2.30.0 and deeptools multiBigwigSummary v3.5.1 were used to identify putative enhancer. Briefly, for each tissue EGFP+ H3K27ac MACS2 narrowPeak peaks were intersected with EGFP+ ATAC peaks present in both duplicates and extended by 150bp on each side. ChIP peaks of interest were then filtered against a -2kb/+500b window centered at transcription start sites of protein coding genes to exclude promoters and proximal cis regulatory elements. Coverage on filtered ChIP peaks of interest was computed with deepTools multiBigwigSummary for the normalized coverage of H3K27ac ChIP of EGFP+ and EGFP-. Putative chondrogenic enhancers were then called as intervals displaying a fold change of normalized H3K27ac coverage in EGFP+/EGFP- ≧4 and an EGFP+ normalized H3K27ac ChIP coverage ≧0.5 and were then merged within 500bp using Bedtools merge. A reciprocal analysis was followed to identify non-chondrogenic enhancers (EGFP-/EGFP+ ≧4 and an EGFP- normalized H3K27ac ChIP coverage ≧0.5). Chondrogenic and non-chondrogenic enhancers are listed respectively in Supplementary Table 5 and 6. To determine chondrogenic enhancer tissue specificity (Supplementary Table 5), the chondrogenic enhancers identified in each tissue were aggregated (overlapping peaks were merged). The normalized H3K27ac coverage was computed with deepTools multiBigwigSummary and a two-fold enrichment between coverages was used as a threshold to characterize these peaks into limb-enriched, trunk-enriched, and pan-chondrogenic enhancers. Chondrogenic enhancers were lifted from mm39 to hg38 using the UCSC liftover tool (https://genome.ucsc.edu/cgi-bin/hgLiftOver) (Supplementary Table 11).

7- Categorization of ChondroTADs and ChondroEnhTADs.
Raw reads from Hi-C datasets generated from E14.5 mouse forelimb cartilage were downloaded from SRA (SRP339920) and mapped to mm39 using HiCUP v0.8.1 , Samtools v.1.10 and Cooler v0.9.3 . The BAM file was then converted to a tabular file with a Python script (https://github.com/lldelisle/tools-lldelisle/blob/8ac44b0341c70ce330fc0f24712b6f9b59b14731/tools/fromHicupToJuicebox/fromHicupToJuicebox.py). The mapped read pairs were then loaded to 20-kb resolution matrices with Cooler makebins. The two raw matrices

replicates were merged using HiCExplorer v3.7.2 (99, 103) hicSumMatrices and normalized with Cooler balance --cis-only. TADs were then called using hicFindTADs --minDepth 650000 --maxDepth 1300000 --step 1300000. Overlap between chondrogenic enhancers, protein-coding chondrogenic genes and TADs was performed using the R packages GenomicRanges v1.48.0 and plyranges v1.16.0 . ChondroTADs and chondroEnhTADs coordinates are listed in Supplementary Table 13 and 14.

8- Gene Ontology enrichment analysis. Protein-coding genes located in chondroTAD (Supplementary Table 15) and chondroEnhTAD (Supplementary Table 16) were analyzed for GO biological process enrichment using the PANTHER Overrepresentation Test (Released 20231017) using the website http://geneontology.org .

Motif enrichment analysis. We performed our motif enrichment analyses using the R package motifcounter v1.18.0 (33).This method relies on a higher-order Markov background model to compute the expected motif hits and a compound Poisson approximation for testing the motif enrichment compared to the chosen background. We use the default parameters for the order of the background model (order=1) and the false-positive level for motif hits (α=0.001). In our analysis, we apply the method to two sets of genomic regions, (1) chondrogenic enhancers and (2) accessible regions without H3K27ac signal ("inactive"). In both sets, the genomic regions were centered at the corresponding ATAC-seq peaks. The same genomic region sets were used as background, respectively. After obtaining the enrichment scores for both sets, we refer to the log2 fold-change value for the over- or underrepresentation of a motif in the chondrogenic enhancers compared to the "inactive" regions. All genomic regions were reduced to a length of 500 bp before the analysis. We tested for enrichment of the binding profiles of 356 TFs in total which were downloaded from the HOCOMOCO database (mouse core collection, v11, mononucleotide PCMs) .

Heatmap visualizations. Read signals for ChIP-seq and ATAC-seq data were visualized with the plotHeatmap function of deepTools v3.5.1. The represented genomic regions were centered on the corresponding ATAC-seq peaks, where proximal peaks (center less than 75bp apart) were merged to avoid redundancy, if indicated in figure caption.

9- Comparisons with published experiments

Mouse SOX9 ChIP-seq. SOX9 ChIP-seq fastq reads were obtained from and reprocessed using the analysis pipeline described above. MACS2 narrowPeak peaks were merged within 500bp and then overlapped with our chondrogenic enhancer set using Bedtools merge and intersect v2.30.0 .

Mouse ATAC-seq: ATAC-seq fastq reads were obtained for E9.75, E10.5 and E11.5 from and for E11.5 to E14.5 from and reprocessed using the analysis pipeline described above. The latter datasets were produced by the Axel Visel and Len Pennacchio laboratory, LBNL with following identifiers ENCSR377YDY (E11.5), ENCSR551WBK (E12.5), ENCSR896XIN (E13.5), ENCSR460BUL (E14.5).

Mouse Longshanks. We lifted the TAD spans of the eight discrete murine Longshanks loci reported by Castro et al. from mm10 to mm39 using the UCSC liftover tool (https://genome.ucsc.edu/cgi-bin/hgLiftOver). Overlap between these eight regions and our chondrogenic enhancers and chondrogenic protein-coding genes was then performed using the R packages GenomicRanges v1.48.0 and plyranges v1.16.0 . The circos plot was produced using the R package circlize v0.4.15 .

Human GWAS height. We analyzed the overlap of our enhancer sets with published human GWS-loci, which were defined as non-overlapping genomic segments that contain at least one quasi-independent genome-wide significant (GWS) SNP associated to adult human height as well as common SNPs from the HapMap3 project in the close vicinity of GWS SNPs. We lifted 7'209 GWS-loci from the EUR cohort from hg19 to mm10 using the UCSC liftover tool (https://genome.ucsc.edu/cgi-bin/hgLiftOver), resulting in 6'926 GWS loci (283 conversions failed). Next, we lifted these regions over to mm39, resulting in the final 6'916 GWS loci we used for our overlap analyses (10 conversions failed). For some analyses, we split the GWS loci into "protein-coding" and a "non-protein-coding" GWS loci based on their overlap with 21'614 protein-coding genes obtained from a filtered GTF file based on ENSEMBL GRCm39 release 104 (see Genomic data section for details). We found 5'145 GWS loci overlapping with at least one protein-coding gene, and 1'771 GWS loci without overlap. For each GWS-loci, we also knew the genetic variance of height explained by GWS SNPs within the loci. The overlap analysis of enhancers and GWS-loci was done using the findOverlaps function of the R package GenomicRanges v1.38.0 . Specifically, we checked for each GWS-loci in an iterative manner, starting from the one with the highest variance explained, if there are any overlapping enhancers. Then, we assign the corresponding variance to the overlapping enhancer(s) and plot the cumulative variances of GWS-loci (x-axis) against the corresponding cumulative variance that can be explained by overlapping enhancers (y-axis). The enhancer sets were matched in size by sorting enhancers based on the maximum H3K27ac enrichment in limb or trunk, and considering only the top 877 enhancers.

For manuscripts utilizing custom algorithms or software that are central to the research but not yet described in published literature, software must be made available to editors and reviewers. We strongly encourage code deposition in a community repository (e.g. GitHub). See the Nature Portfolio guidelines for submitting code & software for further information.

Data

Policy information about availability of data

All manuscripts must include a data availability statement. This statement should provide the following information, where applicable:

- Accession codes, unique identifiers, or web links for publicly available datasets
- A description of any restrictions on data availability
- For clinical datasets or third party data, please ensure that the statement adheres to our policy

Sequencing data are available in the GEO repository under the accession number GSE230235 [https://www.ncbi.nlm.nih.gov/geo/query/acc.cgi?acc=GSE230235]. E13.5 SOX9 ChIP-seq was obtained from [https://www.ncbi.nlm.nih.gov/biosample/SAMD00028628]. Embryonic limb ATAC-seq datasets were obtained from GEO repository [https://www.ncbi.nlm.nih.gov/geo/query/acc.cgi?acc=GSE164738] and the SRA website [https://www.ncbi.nlm.nih.gov/sra/] with the following accession numbers: SRR14305872, SRR14305873, SRR14305249, SRR14305250, SRR14305866, SRR14305867, SRR14306149, SRR14306150, SRR14306037 and SRR14306038. The customized GRCm39/mm39_eGFP-SV40pA sequence and the corresponding filtered GTF file are available on Zenodo [https://doi.org/10.5281/zenodo.7837435]. Source data are provided with this paper.

Research involving human participants, their data, or biological material

Policy information about studies with human participants or human data. See also policy information about sex, gender (identity/presentation), and sexual orientation and race, ethnicity and racism.

Reporting on sex and gender	N/A

Reporting on race, ethnicity, or other socially relevant groupings	N/A
Population characteristics	N/A
Recruitment	N/A
Ethics oversight	N/A

Note that full information on the approval of the study protocol must also be provided in the manuscript.

Field-specific reporting

Please select the one below that is the best fit for your research. If you are not sure, read the appropriate sections before making your selection.

[x] Life sciences [ ] Behavioural & social sciences [ ] Ecological, evolutionary & environmental sciences

For a reference copy of the document with all sections, see nature.com/documents/nr-reporting-summary-flat.pdf

Life sciences study design

All studies must disclose on these points even when the disclosure is negative.

Sample size	For scRNA-seq, sample size is a singlicate of 6 micro-dissected limb pairs. For flow-cytometry experiments, at least 100k cells were recorded by Cytoflex per replicate (2-4 limbs or trunk replicates). Sample size for bulk sampling of RNA-seq was obtained from 4 replicates of 1-2 limb pairs and 1 trunk.<br>The minimal number of FACS sorted or unsorted cells for all experiments, and thus the number of pooled limb pairs, are determined by the type of experiments (RNA-seq: 150K cells, ChIP-seq: 500K cells, ATAC-seq: 75k cells). ATAC-seq and RNA-seq experiments from sorted cells were performed in 2 replicates from pools of 2-3 limbs/trunks. For H3K27ac ChIP-seq experiments were performed from singlicates of 2-4 FACS-sorted pooled limb/trunks. These are commonly accepted sample size for these types of experiments. Moreover, each replicate result is an average of measurements across many pooled samples.<br>For fluorescence microscopy experiments, at least three E14.5 fetus were checked but only one was imaged by light-sheet.
Data exclusions	No data was excluded from this study.
Replication	Experiments were performed in replicates when the results were likely to have a significant impact on the interpretation of the results.<br>FACS-sorted RNA-seq and ATAC-seq were performed in duplicates and bulk RNA-seq in quadruplicates.<br>For scRNA-seq experiment, a single library was build.<br>H3K27ac ChIP-seq experiments were performed in singlicates.<br>Cytoflex flow-cytometry analyses were done in quadruplicates, the signal was pooled for graphical representation.<br>Bulk RNA-seq of wildtype and mutant limbs/trunk were performed in quadruplicates.<br>Light-sheet fluorescence was imaged in singlicate.<br>All attempts at replication were successful.
Randomization	In this work, it was necessary to know the genotypes and types of analyzed cells as they needed to be pooled prior to processing for experiments.
Blinding	Investigators were not blinded since micro-dissection of mouse embryos and further analyses require knowledge about the genotypes and type of cells (EGFP+/-) at hand.

Reporting for specific materials, systems and methods

We require information from authors about some types of materials, experimental systems and methods used in many studies. Here, indicate whether each material, system or method listed is relevant to your study. If you are not sure if a list item applies to your research, read the appropriate section before selecting a response.

Materials & experimental systems

n/a	Involved in the study	
[ ]	[x]	Antibodies
[ ]	[x]	Eukaryotic cell lines
[x]	[ ]	Palaeontology and archaeology
[ ]	[x]	Animals and other organisms
[x]	[ ]	Clinical data
[x]	[ ]	Dual use research of concern
[x]	[ ]	Plants

Methods

n/a	Involved in the study	
[ ]	[x]	ChIP-seq
[ ]	[x]	Flow cytometry
[x]	[ ]	MRI-based neuroimaging

Antibodies

Antibodies used	Polyclonal rabbit anti-H3K27ac antibody from Diagenode, Cat-No. c15410174, was used at a dilution of 1/500.
Validation	The antibody was validated for ChIP usage on the manufacturer's website: https://www.diagenode.com/en/p/h3k27ac-polyclonal-antibody-classic-50-mg-42-ml. Moreover, we extensively used the same antibody in a prior study and observed a high reproducibility.

Eukaryotic cell lines

Policy information about cell lines and Sex and Gender in Research

Cell line source(s)	Male G4 mouse ESCs (Jorge et al., 2007)
Authentication	Genetically modified pluripotent mouse ESCs were authenticated by the production of fetuses through tetraploid aggregations (Artus and Hadjantonakis 2011) and further genotyping confirmed the presence of the desired mutations in the cells.
Mycoplasma contamination	All cell lines tested negative for mycoplasma contamination.
Commonly misidentified lines (See ICLAC register)	No commonly misidentied cell lines were used.

Animals and other research organisms

Policy information about studies involving animals; ARRIVE guidelines recommended for reporting animal research, and Sex and Gender in Research

Laboratory animals	For aggregations: Fetuses were generated from male G4 mouse ESCs (Jorge et al., 2007) via tetraploid complementation (Artus and Hadjantonakis 2011). Donor male and female tetraploid embryos were provided from in vitro fertilisation using C57Bl6J x B6D2F1 backgrounds. Aggregated embryos were transfered into CD1 foster females and micro-dissected at embryonic stage E14.5. Three- to twelve-month-old foster females were housed under standard conditions before sacrifice.<br><br>For enhancer assay: Transgenic E14.5 mouse fetuses were generated as described previously (Osterwalder et al., 2022). Briefly, super-ovulating female FVB mice were mated with FVB males and fertilized embryos were collected from the oviducts. Regulatory element sequences (Supplementary Data 21) were amplified from human genomic DNA or synthesized (Twist Biosciences) and cloned into the donor plasmid containing a minimal β-globin promoter, lacZ reporter gene and H11 locus homology arms using NEBuilder HiFi DNA Assembly Mix (NEB, E2621). The sequence identity of donor plasmids was verified using Nanopore sequencing (Primordium Labs). Plasmids are available upon request. A mixture of Cas9 protein (Alt-R SpCas9 Nuclease V3, IDT, Cat#1081058, final concentration 20 ng/μL), hybridized sgRNA against H11 locus (Alt-R CRISPR-Cas9 tracrRNA, IDT, cat#1072532 and Alt-R CRISPR-Cas9 locus targeting crRNA, gctgatggaacaggtaacaa, total final concentration 50 ng/μL) and donor plasmid (12.5 ng/μL) was injected into the pronucleus of donor FVB embryos. The efficiency of targeting and the gRNA selection process is described in detail in Osterwalder et al., 2022. Embryos were cultured in M16 with amino acids at 37oC, 5% CO2 for 2 hours and implanted into pseudopregnant CD-1 mice. Fetuses were collected at E14.5 for lacZ staining as described previously (Osterwalder et al., 2022). Briefly, fetuses were dissected from the uterine horns, washed in cold PBS, fixed in 4% PFA for 30 min and washed three times in embryo wash buffer (2 mM MgCl2, 0.02% NP-40, and 0.01% deoxycholate in PBS at pH 7.3). They were subsequently stained overnight at room temperature in X-gal stain (4 mM potassium ferricyanide, 4 mM potassium ferrocyanide, 1 mg/mL X-gal and 20 mM Tris pH 7.5 in embryo wash buffer). PCR using genomic DNA extracted from embryonic sacs digested with DirectPCR Lysis Reagent (Viagen, 301-C) containing Proteinase K (final concentration 6 U/mL) was used to confirm integration at the H11 locus and test for presence of tandem insertions (Osterwalder et al., 2022). Only fetuses with donor plasmid insertion at H11 were used. Fetuses of both sexes were used in the analysis. The stained transgenic fetuses were washed three times in PBS and imaged from both sides using a Leica MZ16 microscope and Leica DFC420 digital camera. All images are in the Vista enhancer browser (https://enhancer.lbl.gov/) (Visel et al., 2007) with the hs numbers 2696, 2697, 2698 and 2700.
Wild animals	There was no wild animals in this study.
Reporting on sex	Sex was not part of the study design. Yet, for aggregations: fetuses were generated from male G4 mouse ESCs (Jorge et al., 2007) and therefore are males. For enhancer assay: fetuses of both sexes were used in the analysis.
Field-collected samples	There was no field collected animals in this study.
Ethics oversight	Animal procedures performed in Geneva were in accordance with institutional, state, and government regulations (Canton de Genève authorizations GE/89/19 and GE192A). Animal work performed at Lawrence Berkeley National Laboratory (LBNL) was reviewed and approved by the LBNL Animal Welfare Committee under protocol numbers #290003 and #290008.

Note that full information on the approval of the study protocol must also be provided in the manuscript.

Plants

Seed stocks	*Report on the source of all seed stocks or other plant material used. If applicable, state the seed stock centre and catalogue number. If plant specimens were collected from the field, describe the collection location, date and sampling procedures.*
Novel plant genotypes	*Describe the methods by which all novel plant genotypes were produced. This includes those generated by transgenic approaches, gene editing, chemical/radiation-based mutagenesis and hybridization. For transgenic lines, describe the transformation method, the number of independent lines analyzed and the generation upon which experiments were performed. For gene-edited lines, describe the editor used, the endogenous sequence targeted for editing, the targeting guide RNA sequence (if applicable) and how the editor was applied.*
Authentication	*Describe any authentication procedures for each seed stock used or novel genotype generated. Describe any experiments used to assess the effect of a mutation and, where applicable, how potential secondary effects (e.g. second site T-DNA insertions, mosiacism, off-target gene editing) were examined.*

ChIP-seq

Data deposition

☒ Confirm that both raw and final processed data have been deposited in a public database such as GEO.

☒ Confirm that you have deposited or provided access to graph files (e.g. BED files) for the called peaks.

Data access links<br>*May remain private before publication.*	https://www.ncbi.nlm.nih.gov/geo/query/acc.cgi?acc=GSE230235
Files in database submission	H3K27ac-L-E145-Col2a1GFP-GFPp-R1_S6_L008_R1_001.fastq.gz<br>H3K27ac-L-E145-Col2a1GFP-GFPn-R1_S5_L008_R1_001.fastq.gz<br>H3K27ac-T-E145-Col2a1GFP-GFPp-R1_S29_L007_R1_001.fastq.gz<br>H3K27ac-T-E145-Col2a1GFP-GFPn-R1_S28_L007_R1_001.fastq.gz<br>H3K27ac-L-E145-Col2a1GFP-GFPp-R1_S6_L008_macs_SR200_peaks.narrowPeak<br>H3K27ac-L-E145-Col2a1GFP-GFPn-R1_S5_L008_macs_SR200_peaks.narrowPeak<br>H3K27ac-T-E145-Col2a1GFP-GFPp-R1_S29_L007_macs_SR200_peaks.narrowPeak<br>H3K27ac-T-E145-Col2a1GFP-GFPn-R1_S28_L007_macs_SR200_peaks.narrowPeak<br>H3K27ac-L-E145-Col2a1GFP-GFPp-R1_S6_L008_macs_SR200_norm.bw<br>H3K27ac-L-E145-Col2a1GFP-GFPn-R1_S5_L008_macs_SR200_norm.bw<br>H3K27ac-T-E145-Col2a1GFP-GFPp-R1_S29_L007_macs_SR200_norm.bw<br>H3K27ac-T-E145-Col2a1GFP-GFPn-R1_S28_L007_macs_SR200_norm.bw
Genome browser session<br>(e.g. UCSC)	Not applicable

Methodology

Replicates	ChIP-seq were performed in singlicates from FACS-sorted cells obtained from pooled pairs of limb and trunks.
Sequencing depth	A similar number of reads (39x10e6) were randomly sampled from each ChIP-seq dataset to correct for sequencing depth variation using Seqtk v1.3 (-s 100) (https://github.com/lh3/seqtk).
Antibodies	H3K27Ac ChIP-seq was performed using C15410174 (Diagenode) with 1/500 dilution of the antibody.
Peak calling parameters	Peak calling was obtained after extension of the reads by 200 bp using MACS2 v2.2.7.1 (callpeak --nomodel --call-summits --extsize 200).
Data quality	Data quality was manually confirmed by observation by the observation of a strong enrichment of H3K27ac signal at known chondrogenic and non-chondrogenic loci, such as the Hox clusters.
Software	A similar number of reads (39x10e6) were randomly sampled from each ChIP-seq dataset to correct for sequencing depth variation using Seqtk v1.3 (-s 100) (https://github.com/lh3/seqtk). TruSeq adapter sequences and bad quality bases were removed using CutAdapt v1.18 (-a AGATCGGAAGAGCACACGTCTGAACTCCAGTCAC -q30 -m15). Reads were then mapped to GRCm39/mm39 using Bowtie2 v2.3.5.1 with default parameters. Reads were then filtered for a MAPQ≥30 using Samtools view v1.10 and the coverage and peak calling was obtained after extension of the reads by 200 bp using MACS2 v2.2.7.1 (94) (callpeak --nomodel --call-summits --extsize 200). Coverage was normalized by the number of million tags used by MACS2.

Flow Cytometry

Plots

Confirm that:

- [x] The axis labels state the marker and fluorochrome used (e.g. CD4-FITC).
- [x] The axis scales are clearly visible. Include numbers along axes only for bottom left plot of group (a 'group' is an analysis of identical markers).
- [x] All plots are contour plots with outliers or pseudocolor plots.
- [x] A numerical value for number of cells or percentage (with statistics) is provided.

Methodology

Sample preparation	Tissues were prepared following a similar protocol for flow-cytometry analysis, FACS-sorting for RNA-seq, ChIP-seq and ATAC-seq and single-cell RNA-seq. Briefly, limbs and trunk samples were dissected from decapitated E14.5 embryos in cold PBS solution. Tissues were first minced using a pair of micro-scissors. After PBS removal, a single cell suspension was achieved by incubating tissues in 1.2mL Trypsin-EDTA (Thermo Fischer Scientific, 25300062) for 15' to 18' at 37°C in a thermomixer with resuspension steps each 6'. After blocking with one volume of 5% BSA (Sigma Aldrich, A7906-100G), cells were passed through a 40µm cell strainer for further tissue disruption and another volume of 5% BSA was added to the cell strainer to pass leftover cells. Cells were then centrifuged at 400×g for 5' at 4°C and, after discarding the supernatant, they were resuspended in 1% BSA for cell sorting. 5mM of Na-Butyrate were added to the BSA when planning for subsequent fixation for H3K27Ac-ChIP.
Instrument	BD FACSAria Fusion and Beckman Coulter CytoFLEX
Software	Data was formatted using FlowJo v10.9.
Cell population abundance	Borders to determine population abundance are displayed in main and supplemental figures
Gating strategy	For FACS sorting, cell populations were isolated using the BD FACSAria Fusion with a 488nm laser and a 530/30 filter for GFP. Initial FSC/SCC was set between 30/40 and 210/240 to exclude debris. After removal of dead cells with Draq7 dye and removal of doublets, following standard protocol, cells were gated for sorting.<br><br>Bulk analysis of GFP signal was quantified with a Beckman Coulter CytoFLEX flow-cytometer using a 488nm laser and a 525/40 filter. Single cells were identified based on their FSC/SCC features.

- [x] Tick this box to confirm that a figure exemplifying the gating strategy is provided in the Supplementary Information.
